# Annotations

**Published:** 1893-12-30

**Authors:** 


### Annotations.

### "Ecclesiastical Evolution."

The medical writer may not ordinarily have much connection with things ecclesiastical, but he has of necessity a very great deal to do with things evolutionary. In reviewing the life of the late Dean Stanley, just published, the *Times* used the significant phrase, "Ecclesiastical Evolution." "Dogmatic orthodoxy," says the reviewer, "respect for ecclesiastical tradition, reverence for the teaching of antiquity, are the dominant notes of Pusey's ecclesiastical temper. Stanley, on the other hand, was, as it were, the apostle of ecclesiastical evolution." Few things show better than this the hold which biology, physiology, and the temper of modern science has obtained upon the modern mind. The critical, explanatory, expectant, and progressive attitude of mind which is so characteristic of science in general, and of the sciences which associate themselves with medicine in particular, is gradually pervading every department of thought and life, alike in State and Church. Our object in calling attention to this question here and elsewhere is that we may remind medical men and others who pursue the ancillary medical sciences of this undoubted phenomenon which is now everywhere manifest, and of the corresponding obligations which rest upon those whose labours have brought about the universal predominance of the phenomenon. If evolution is the leading idea of modern times, it follows that evolutionists should be the leading persons. But evolutionists cannot lead by virtue of their knowledge of evolution, but only by virtue of their practical capacity for leadership. The soldier is not a successful general because he carries Her Majesty's commission in his pocket, but because he has the innate capacity for disposing an army and leading it to victory. Evolutionists, if they would secure for the world all the fruitful progress which is latent in the doctrine of evolution, must study not evolution only, but evolution and man. They will then, like Dean Stanley, see the golden age in the future, not in the past; they will see it, and they will be the immortal pioneers who will bring it in.

### Diet for Diabetics.

Diabetics, pronounced and in the making, are many; and to them almost the most important thing in life is the daily diet. Diet and quiet are the sheet-anchors of their well-being. Dr. Saundby, of Birmingham, has called attention to the value and palatability of Iceland moss for puddings and blanc manges. It may not be very generally known that Irish moss, which is practically identical with the Iceland variety, is very cheap, and makes delicious puddings and blanc manges, that are in every way suitable. Gluten bread, Dr. Saundby says, he is fast giving up, even the best varieties. The sham substitutes sold under the name of "Gluten" are wicked delusions. Almond cakes our diabetic mentor still continues to believe in; and certainly for the diabetic in every stage of his progress they are of undoubted value. This is Dr. Saundby's excellent recipe for almond cakes: "One pound of ground almonds, four eggs, two tablespoonfuls of milk, and a pinch of salt; beat up the eggs and milk, and stir in the almond flour; divide in twelve flat tins, and bake in a moderate oven for about forty-five minutes." This, it will be seen, promises, not only a highly nutritious, but a very palatable cake. Diabetes is one of those diseases which emphatically demand the intelligent co-operation of the patient. The two things which the patient can mostly do for himself are the cultivation of a philosophic frame of mind, and the training of the palate to rigid medical obedience.

### Complaints against Country Hopitals.

Two letters which appeared in the *Oxford Journal* of December 2nd, demand more than a passing consideration. The Radcliffe Infirmary at Oxford announces to the public the name of a certain well-known gentleman as physician to out-patients. The public who subscribe and the patients who attend naturally expect that that physician will see out-patients, and will prescribe for them, as he is bound to do. But if the writers in the *Oxford Journal* are to be trusted, that physician does not seem to attend to his announced duties systematically, but leaves the out-patients, to the inexperience of the newly qualified house physician. One or two facts reported by "Clericus" and "Oxonian" will illustrate the position. "It has several times happened to me," writes "Clericus," "when my parishioners have become out-patients, to ask them after having been to Oxford: 'What does your doctor say of you?' and then to have this answer: 'I did not see him, I only saw the house doctor.' We, sir, who are subscribers, expect that the medical officer whose name appears as responsible . . . should personally acquit himself of that responsibility." "Clericus" is surely right in this protest. "Oxonian" writes that an out-patient informed him she had attended weekly for seven weeks before she could see the physician under whose care she was stated on her card to be. He relates the case of another out-patient who attended and saw only the house doctor, whose diagnosis proved incorrect. One more fact. The physician in question is stated to receive the following sums annually from the Radcliffe: £79 from Dr. Frewin's trust, £20 for lectures to nurses, £100 for clinical lectures from the Lichfield trust; in all, £199. "Oxonian" adds that "for several years past the decadence of the Infirmary in public estimation has been very marked." Both subscribers and patients have fallen off in large numbers. The subscription income in 1885 was £3,025, whereas last year it was only £2,714. The out-patients last year were nearly 2,000 fewer than in 1891. It is fair to say that we have heard but one side of the question. There is another side which the public would be glad to hear. It is also proper to add that country hospitals and their physicians and surgeons are the subjects of many unreasonable complaints on the part, especially, of out-patients, and that clerical and other critics are not unwilling to have a fling at them on the smallest provocation. The public, therefore, whilst noting what has been said, will do well to wait until it can be furnished with an account of the other side of the question.

